# 

**Published:** 1914-02

**Authors:** 


*OFFICIAL ORGAN—State Society Social Hygiene and Eugenics.*

VOL. XXIX FEBRUARY, 1914 No. 8

General, U. S.
ington, D. C.

### The "Red Back"

### TEXAS MEDICAL JOURNAL

ESTABLISHED JULY, 1885

PUBLISHED MONTHLY AT AUSTIN, TEXAS, BY

F. E. DANIEL, M. D., - - - Editor and Proprietor.

PRINTED BY THE VON BOECKMANN-JONES CO.

Subscription, $1.00 a Year, in Advance. - - - - - Single Copies, 10 Cents

